# DDAO Controlled Synthesis of Organo-Modified Silica Nanoparticles with Encapsulated Fluorescent Boron Dipyrrins and Study of Their Uptake by Cancerous Cells

**DOI:** 10.3390/molecules25173802

**Published:** 2020-08-21

**Authors:** Aleksandr A. Goncharenko, Ilya A. Tarasyuk, Yuriy S. Marfin, Kirill V. Grzhegorzhevskii, Albert R. Muslimov, Andrey B. Bondarenko, Maxim D. Lebedev, Ilya A. Kuz’min, Artur S. Vashurin, Kirill V. Lepik, Alexander S. Timin, Evgeniy V. Rumyantsev

**Affiliations:** 1 Department of Biophysics, Polytechnicheskaya, Peter The Great St. Petersburg Polytechnic University, 29, 195251 St. Petersburg, Russia; alexs.goncharenko@yandex.ru (A.A.G.); a_timin@mail.ru (A.S.T.); 2 Ivanovo State University of Chemistry and Technology, Sheremetevsky str., 7, 153000 Ivanovo, Russia; ymarfin@gmail.com (Y.S.M.); maximlebedev37@gmail.com (M.D.L.); wonderful_37@list.ru (I.A.K.); asvashurin@mail.ru (A.S.V.); evr@isuct.ru (E.V.R.); 3 Institute of Natural Sciences and Mathematics, Ural Federal University, 19, 620002 Mira St. Ekaterinburg, Russia; kirillica5@mail.ru; 4 RM Gorbacheva Research Institute of Pediatric Oncology, Hematology and Transplantation, Pavlov University, Lva Tolstogo 6/8, 197022 St. Petersburg, Russia; albert.r.muslimov@gmail.com (A.R.M.); lepikkv@gmail.com (K.V.L.); 5 St. Petersburg State University, 7/9 Universitetskaya nab., 199034 Saint-Petersburg, Russia; bonan@bk.ru; 6 Research School of Chemical and Biomedical Engineering, National Research Tomsk Polytechnic University, Lenin Avenue 30, 634050 Tomsk, Russia; 7 Ivanovo State Polytechnic University, Ivanovo, Sheremetevsky str., 21, 153002 Ivanovo, Russia

**Keywords:** silica nanoparticles, soft-template, oil-in-water, one-pot synthesis, BODIPY

## Abstract

The design of cargo carriers with high biocompatibility, unique morphological characteristics, and capability of strong bonding of fluorescent dye is highly important for the development of a platform for smart imaging and diagnostics. In this paper, BODIPY-doped silica nanoparticles were prepared through a “one-pot” soft-template method using a sol-gel process. Several sol-gel precursors have been used in sol-gel synthesis in the presence of soft-template to obtain the silica-based materials with the most appropriate morphological features for the immobilization of BODIPY molecules. Obtained silica particles have been shown to be non-cytotoxic and can be effectively internalized into the cervical cancer cell line (HeLa). The described method of synthesis allows us to obtain silica-based carriers with an immobilized fluorescent dye that provide the possibility for real-time imaging and detection of these carriers.

## 1. Introduction

The chemotherapeutic delivery systems, such as polymer conjugates [1,2], micelles [3], liposomes [4,5], dendrimers [6], and organic/inorganic hybrid nanoparticles [7], have been widely explored as diagnostic devices in cancer research. Despite the recent progress in the study of nanoparticles used for cancer diagnosis, visualization of tumor tissues with sufficient sensitivity, spatial resolution, and unique stability is still actual. 

In the case of fluorescence-based sensing and bio-imaging applications for living systems, it is highly desirable to develop fluorophores with high quantum yield and light stability [8]. Many fluorescent dyes are currently using for biomedical imaging analysis [9], such as cyanine dyes [10], tetrapyrrole dyes [11], thiazide/oxazine dyes [12]. However, they have several disadvantages, associated with poor light stability, small Stokes shift, and poor water solubility. Boron dipyrrins (BODIPYs) have attracted much attention as fluorescent agents due to their narrow fluorescence peaks, high quantum yields, long-wavelength emission, and good stability in physiological conditions [9]. Moreover, the variety of chemical structure of BODIPY molecules makes them promising for functionalization of different drug carriers. In this case, the functionalization of drug carriers using BODIPY dyes provides an excellent opportunity to develop therapeutic nanoplatforms that possess high fluorescence quantum yields for in vitro and in vivo imaging. BODIPY dyes in this case are good model compounds allowing to demonstrate the features and risks of dye immobilization and allowing to move further to the new functional dye classes and groups.

Among the diversity of drug carriers, silica hybrid nanoparticles prepared by the sol-gel technique has been demonstrated as a unique tool for in situ encapsulation of different drugs and bioactive compounds [13]. The sol-gel chemistry provides an excellent opportunity to fabricate hybrid silica nanoparticles with novel physicochemical features, including a low permeability and intracellular degradation. Mesoporous silica nano/microspheres have been shown to be good materials as cargo carriers, because of the particular characteristics such as large specific surface area, pore volume and pore size [14]. Nowadays many researchers focus on the application of silica-based materials in nanomedicine [15,16]. To obtain silica particles with core-shell structures and required parameters, different methods of template synthesis are used [17,18]. Two main synthetic approaches, hard-templating and soft-templating methods, are broadly employing for the synthesis of silica particles with hollow or core-shell structures [19]. 

As for the hard-templating method, solid particles are applied as “hard” core templates and silica shell is formed on the surface of solid particles during sol-gel synthesis [20,21]. The materials prepared by the hard template method without the application of surfactants have low specific surface area [22]. The main disadvantage of the hard template method is that solid templates are removed by calcination (high temperature treatment) or aggressive chemicals [23], which makes it unable to perform in situ cargo encapsulation.

The essence of the soft-templating method is that the synthesis could be performed in the presence of organic surfactants in water-in-oil (W/O) emulsion [19]. In the emulsion system, the organic component plays an important role in controlling the morphology of the final mesoporous structure and the droplets of water serve as a “soft” template. Emulsions [24], vesicular [25], and micellar [26] structures are usually considered as soft templates. Usually the templates can be removed by a simple washing procedure. They are more attractive due to the possibility to obtain the materials with high specific surface area, the wide capability to modify the particle surface with different functional groups. Employing W/O emulsion approach allows entrapment of cargo inside the final solid particles in a one-pot synthesis. However, strict control of reaction conditions is generally required, because soft templates are very sensitive to the reaction environment [19]. Many studies have been performed using cetyltrimethylammonium bromide (CTAB) [27,28,29] 

The application of nanoemulsions as a soft template allows to perform in situ encapsulation of cargo molecules with high stability. During sol-gel synthesis, hydrolysis and polycondensation of alkoxysilanes occur, forming solid nanoparticles, which will grow around the surface of the micelles [30]. The W/O system is suitable for the fixation of polar components inside the silica particle [31] while the oil-in-water (O/W) emulsion is desirable for nonpolar substances [32]. Nonpolar molecules immobilization is possible due to the hydrophobic effect using the silica matrix modified with phenyl fragments [33,34,35]. These templates can be used only for one-pot immobilization of polar molecules. However, the anchoring of nonpolar molecules such as BODIPY into silica nanoparticles is a technical challenge. Moreover, the “one-pot” method seems like the simplest way for the synthesis of hybrid silica nanoparticles [36,37], which can be used as a carrier of nonpolar components. 

A wide variety of sol-gel precursors for modified silica synthesis allows to design synthetic procedures for specific tasks in the field of biologically active compounds delivery. Therefore, amino-modified silica found application in genetic therapy and its effectiveness comparable with other delivery systems, such as polycations [38]. Besides effective nucleic acids adsorption, silica surface covered with amino groups is also capable to interact with negatively charged cell membrane that causes increase of cellular uptake compared to unmodified silica particles [39,40]. Free amino groups have “proton sponge” effect, that is the key factor of particles transfer from endosomes to cytoplasm also called “endosomal escape” [41,42]. Silica particles modified by nonpolar octyl and octadecene groups [43], aldehyde [44], thiol [45], carboxyl groups [46], could be applied as delivery systems.

Numerous investigations of silica compatibility with different types of cellular lines reported. The most toxic particles are particles with a diameter range of 4–100 nm, since such particles are able to penetrate to cell nucleus and cause genotoxic effect. Wherein, apoptosis is the common way of cell death (approximately 90%) and only 10% of cells dies by necroptosis. Common reasons of cell death are high particles genotoxicity, time and dose-dependent oxidative stress on submicron particles [47]. Investigations of silica surface modification shows that surface amino groups does not change overall silica toxicity [48,49]. Thus, amino-modified particles with size range 200–500 nm, possessing low toxicity, are very promising as drug delivery systems.

Silica inertness related to its high biocompatibility [50]. Despite it, cells have lack of mechanisms of silicon dioxide destruction, therefore silica degradation rate is lower comparing to polyesters of organic acids (PLA, PLGA, PHB etc.), polyanions (dextral sulfate, PEI, polyamino acids), phospholipids, and many other organic compounds traditionally used in the drug delivery. Possible solution for this problem is the modification of structure by enzymatically responsive bonds [51]. Previous investigations showed that cellular uptake of 200–400 nm particles occurred by phagocytic or/and clathrin dependent pathways. This mechanism is belong to both cancerous and healthy cells. Additionally the possible caveolin dependent endocytosis for 200 nm particles on A562 and THP-1 cellular lines was marked [52,53]. Thus, silica particles modification has broad opportunities of bioactive compounds delivery and imparting enzymes sensitivity allows producing carriers with effective intracellular degradation.

Herein we developed a simple “one-pot” O/W method for the preparation of silica nanoparticles conjugated with BODIPY for cell imaging using novel surfactant *N*,*N*-dimethyldodecylamine *N*-oxide (DDAO) as perspective template generating nanoemulsions and fixation of nonpolar molecules. Different sol-gel precursors, such as tetraethoxysilane, tetraethoxysilane aminopropyltriethoxysilane, phenyltrimethoxysilane, mercaptopropyltrimethoxysilane with different molar ratio were used in our synthesis to identify the most appropriate morphological structures, which were examined by electron microscopy. The most appropriate conditions for the fabrication of uniform silica particles with a high immobilization efficiency of BODIPY were achieved. Such conditions allow to produce silica particles with different modifications via the simple procedure, mild conditions and without thermal treatment to prevent the destruction of surface functional groups. The BODIPY-conjugated silica particles demonstrated high uptake efficiency and biocompatibility on the human cervical cancer cell line (HeLa).

## 2. Results and Discussion

### 2.1. The Influence of Sol-Gel Conditions on the Morphology of Final Silica-Based Materials

**Figure 1:**
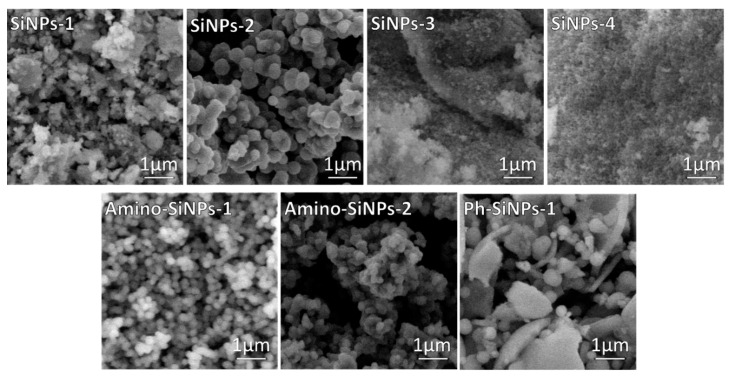
SEM images of silica-based materials obtained by varying sol-gel conditions and organosilanes.

Herein, by varying sol-gel precursors and conditions eight silica samples were obtained. Table S1 in Supplementary Materials describes synthetic conditions and samples designation. Sol-gel synthesis is carried out through hydrolysis reactions of sol-gel precursors in water/ethanol solution to form reactive Si–OH species that condense through dehydration reactions generating three-dimensional silica networks via Si–O–Si bridges. Many factors such as the nature of sol-gel precursor and template used in sol-gel synthesis influence the morphology of final products [19]. To investigate the influence of the template and sol-gel precursors on the process of particle formation, SEM analysis was employed (Figure 1). In the case of unmodified silica samples (**SiNPs-1, SiNPs-2, SiNPs-3, SiNPs-4**), we can clearly observe how the template impact on the morphology of final materials. When sol-gel synthesis was performed in the presence of a template (**SiNPs-1, SiNPs-2**), the nonuniform silica particles are observed. The amorphous agglomerates were detected when sol-gel synthesis was performed without a template (**SiNPs-3, SiNPs-4**). As reported early [38], a template plays an important role in the morphological properties of final materials. The internal hydrophobic region of DDAO allowed the sol-gel precursor in and then provided a place for the precursor to hydrolyze. The sol-gel precursor hydrolyzed to form silica seeds. Since negatively charged, these silica seeds attached to the outer surface of positively charged DDAO via electrostatic interactions, resulting in the formation of uniform spherical silica particles [39].

The reaction rate of TMOS is higher than in the case of TEOS [38]. The low hydrolysis rate limits the particle nucleation and the hydrolyzed species can be aggregated to the larger amorphous structure resulting in lower surface areas. As a result, the addition of TMOS in sol-gel synthesis can yield narrower uniform spherical structures than TEOS (Figure 1, **SiNPs-2**). APTMS and PhTES precursors have been chosen for sol-gel synthesis due to the presence of amino- and phenyl-groups in the chemical structure of these compounds. It allows forming hydrophilic and hydrophobic interactions between silica matrix and organic substances. The SEM images of samples modified with amino- and phenyl-groups indicate that the morphology of final products depends on the type of organosilane precursor used in sol-gel synthesis (Figure 1, **amino-SiNPs-1, amino-SiNPs-2, and Ph-SiNPs-1**). It should be noted that in the case of **amino-SiNPs-1,** when the sol-gel synthesis was performed in the presence of DDAO, the uniform shape and size of silica particles were observed. The SEM image of **amino-SiNPs-2** demonstrated the particles agglomeration, which can be due to the absence of the template in sol-gel synthesis. The SEM image of **Ph-SiNPs-1** demonstrated a significant difference in morphology of final materials. We observed an amorphous mixture of particles and non-uniform agglomerates (sol-gel synthesis together with template—DDAO). When applying the template, uniform spherical nanoparticles were not obtained. The lower rate of hydrolysis and polycondensation of PhTES leads to the formation of big non-uniform clusters of agglomerates. The same observation was previously observed in [40]. It was not able to obtain silica-based material when we performed the sol-gel synthesis using PhTES (**Ph-SiNPs-2)**, but without DDAO (data not shown).

### 2.2. Conjugation of BODIPY-2 to APTMS and Its Spectral Properties

**Figure 2:**
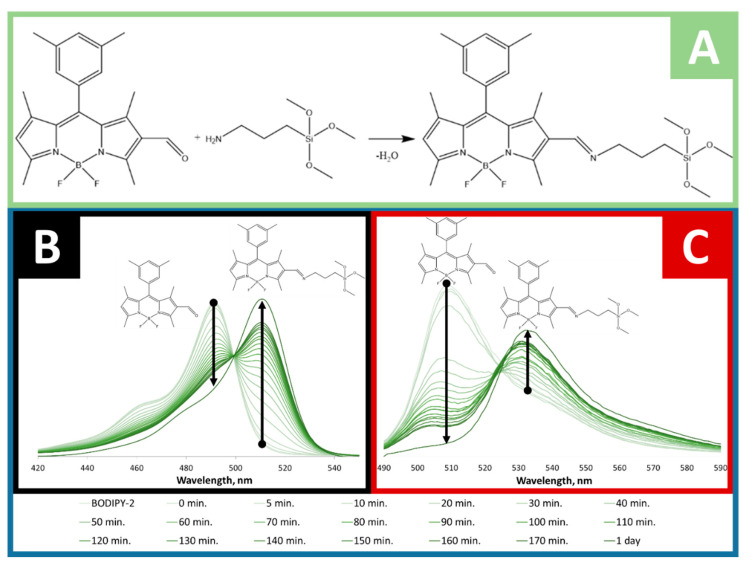
(**A**) Covalent conjugation of APTMS to BODIPY-2 with the formation of azomethine bond. Evolution of absorption (**B**) and fluorescence (**C**) spectra of BODIPY-2 in ethanol solution with APTMS during 1 day.

**Figure 3:**
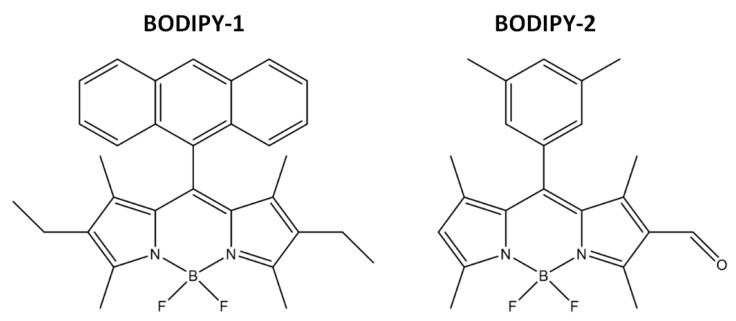
Chemical structures of tested BODIPY dyes.

To find out whether BODIPY-2 was covalently conjugated with APTMS, the small portion absorption and fluorescence spectra were measured during the conjugation process (1 day). The same method of evaluation of the covalent bonding of BODIPY was reported in [39]. The absorption and fluorescence spectra were measured in ethanol solution at room temperature and depicted in Figure 2. BODIPY-2 has an absorption maximum at 492 nm and emission peak at 510 nm. During the conjugation process, the absorption and fluorescence peaks red shifts on 20 nm and 24 nm, respectively (Figure 2B,C). That fact can be attributed to the extension of π-electronic system of BODIPY caused by the formation of azomethine bond (Figure 2A and Figure 3). The obtained sol-gel precursor (APTMS@BODIPY-2) was further used in sol-gel synthesis for the preparation of BODIPY-coated silica-based materials.

### 2.3. Conjugation of BODIPY-1 and BODIPY-2 with Silica-Based Materials and Its Characterization

**Figure 4:**
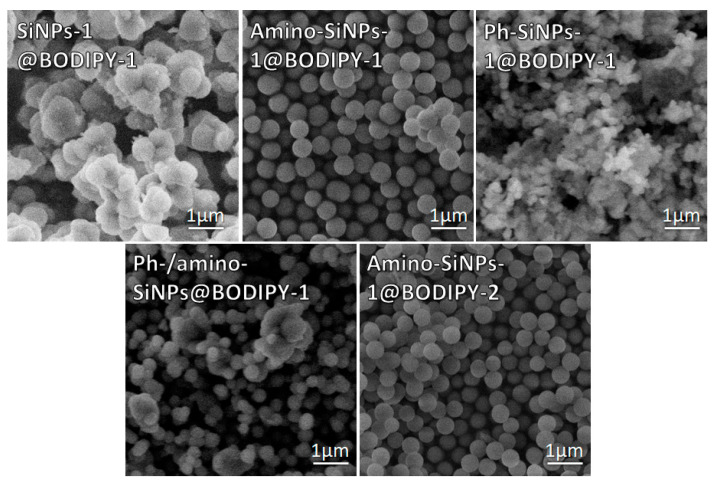
SEM images of BODIPY-doped silica-based materials.

Silica was selected for the immobilization of BODIPY because unlike polymers applied for drug delivery, it does not change porosity or swelling under pH changes. Moreover, silica provides a high biocompatibility and stability in aqueous media. The sol-gel synthesis is very sensitive to any additional components, such as organic molecules [38]. The addition of organic molecules such as BODIPY in sol-gel synthesis may cause strengthening or weakening the hydrogen-bond network of silica matrix, which can lead to structural disorder and morphological changes of final silica-based materials. As we mentioned above, the amino- and phenyl-groups can play a significant role in the immobilization of BODIPY molecules via electrostatic and hydrophobic interaction. Because of that, the immobilization of BODIPY-1 was performed in the presence of APTMS and PhTES. For comparison unmodified silica-based materials was also used for immobilization of BODIPY-1. The **SiNPs-2, amino-SiNPs-1,** and **Ph-SiNPs-1** were selected for further immobilization of BODIPY-1 (Figure 1). The detailed information concerning the synthetic procedure for immobilization of BODIPY-1 is presented in Table S1. The sol-gel synthesis was also performed in the simultaneous presence of APTMS and PhTES (Table S1) to obtain the silica-based materials with amino- and phenyl- functional groups (**Ph-/amino-SiNPs@BODIPY-1)**. Moreover, **APTMS@BODIPY-2** was used in sol-gel synthesis to prepare **amino-SiNPs-1@BODIPY-2**. As a result, five samples were obtained via sol-gel methods with the following abbreviations: **SiNPs-1@BODIPY-1, amino-SiNPs-1@BODIPY-1** and **Ph-SiNPs-1@BODIPY-1**, **Ph-/amino-SiNPs@BODIPY-1,** and **amino-SiNPs-1@BODIPY-2**. The obtained samples were further characterized by SEM in order to reveal the effect of BODIPY on the morphology of silica-based materials. The SEM microphotographs of obtained BODIPY-doped silica-based materials, as shown in Figure 4, demonstrate spherical shape for all tested materials. The **amino-SiNPs-1@BODIPY-1** showed no particle agglomeration and narrow size distribution (400–500 nm). The average size of the individual particles for all tested samples, excepting **amino-SiNPs-1@BODIPY-2** is in the range of 200–600 nm. The particle size of **amino-SiNPs-1@BODIPY-2** is found to be less than 100 nm (Figure 4). The functionalized organosilane such as APTMS@BODIPY-2 has a different rate of hydrolysis and polycondensation compared to APTMS [38]. It has been demonstrated that organo-functionalized silanes are more reactive [41,42] that increases the rate of hydrolysis and leads to induce the growth of nanoparticles. In contrast, PhTES is less reactive [43], and phenyl groups can enhance the competitive condensation reactions resulting in large agglomerates of particles.

### 2.4. FTIR Spectra

**Figure 5:**
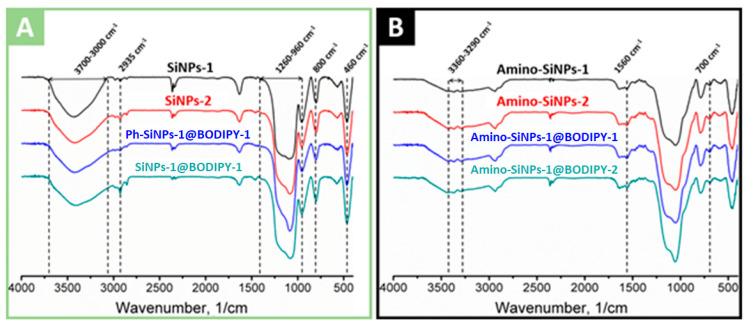
FTIR spectra of (**A**) unmodified and phenyl-modified silica particles (SiNPs-1, SiNPs-2, Ph-SiNPs-1@BODIPY-1, SiNPs-1@BODIPY-1) and (**B**) amino-modified silica particles (amino-SiNPs-1, amino-SiNPs-2, amino-SiNPs-1@BODIPY-1, and amino-SiNPs-1@BODIPY-2).

**Figure 6:**
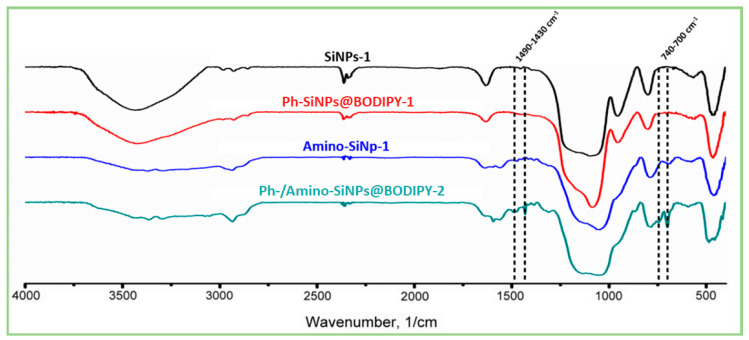
FTIR spectra of SiNPs-1, Ph-SiNPs-1@BODIPY-1, Amino-SiNPs-1, Ph-/amino-SiNPs@BODIPY-1.

The FTIR spectra of unmodified, amino- and phenyl-modified silica samples with immobilized BODIPY are shown in Figure 5A,B, and Figure 6, respectively. Analysis of FTIR spectra of unmodified, phenyl- and amino-modified silica-based materials revealed the characteristic broad absorption band at 3700–3000 cm^−1^, which includes vibrations of surface hydroxyl groups (-OH) of silica. The region at 3000–2800 cm^−1^ is characterized by absorption bands of symmetric and asymmetric C-H vibrations of unreacted alkoxy groups [54]. The band at 2980 cm^−1^ and the weak band at 2910 cm^−1^ correspond to the valence vibrations of the CH_3_ groups, and bands at 2935 cm^−1^ and 2885 cm^−1^ correspond to the valence vibrations of the CH_2_ groups. Several bands at 2400–2300 cm^−1^ are associated with the presence of carbon dioxide in the material. The bands located at 1300–1000 cm^−1^ correspond to asymmetrical vibrations of Si-O-Si (1260–1000 cm^−1^), deformation vibrations of SiO_4_ (1200–850 cm^−1^), valence and deformation vibrations of Si-O (1180–1000 cm^−1^), Si-OC and CO (1260–1000 cm^−1^) vibrations. The band at 960 cm^−1^ corresponds to the stretching vibrations of the silanol groups. Moreover, 800 cm^−1^ is symmetrical Si-O-Si vibrations. The peak in the region of 630–530 cm^−1^ is found in all materials, its appearance is attributed to the influence of short-range alkoxy and aminopropyl groups on the skeletal vibrations of Si-O-Si, which have a characteristic band at 460 cm^−1^. Oscillations corresponding to the aromatic phenyl groups were found only in the case of Ph-/amino-SiNPs@BODIPY-1. The presence of the phenyl groups is proved by bands at 1490, which can be assigned to the aromatic ring vibrations. The sharp weak peaks at 1432 cm^–1^ corresponds to the combination of two vibrations: phenyl C=C stretching vibrations and in-plane C–H deformation [40]. The narrow peaks at 740 cm^–1^ are assigned to the phenyl H out-of-plane ring deformation vibration.

Considering the FITR spectra of amino-modified silica samples, we observe the decrease in the absorption intensity at a region of 3600–3000 cm^−1^ and the complete disappearance of the 960 cm^−1^ band, which attributed to a decrease of hydroxyl groups (−OH). The FTIR spectra of amino-modified silica samples (**amino-SiNPs-1@BODIPY-1**, **amino-SiNPs-1@BODIPY-2**) show asymmetric and symmetric vibrations of the amino group at 3360 cm^−1^ and 3290 cm^−1^, indicating the amino groups were fixed onto silica surface. Strips of valence and deformation vibrations of the CH_2_ group acquire a large intensity, along with the almost total disappearance of CH_3_ oscillations. The peaks at 1595 cm^−1^ and 1560 cm^−1^ are clearly distinguishable, the first is defined as the deformation vibration of the free amino group, the appearance of the second peak, at lower frequencies, is associated with the participation of the amino group in the formation of hydrogen bonds and protonation of the amino group. The vibrations of C-N overlap with the vibrations of the matrix of the bulk phase of silica. The similar FTIR spectra of **amino-SiNPs-1** and **amino-SiNPs-2** confirmed the successful removal of the template (cyclohexane/DDAO) using a washing procedure with ethanol.

Thus, it can be concluded that a sequential modification on the silica surface takes place. It should be noted that the absorption peaks corresponding to functional groups of BODIPY molecules were not detected, it can be associated with low concentration of immobilized BODIPY. Therefore, we further used absorption spectroscopy and fluorescent microscopy to examine the immobilization efficiency of BODIPY molecules.

### 2.5. Study of BODIPY-1 and BODIPY-2 Loading 

**Figure 7:**
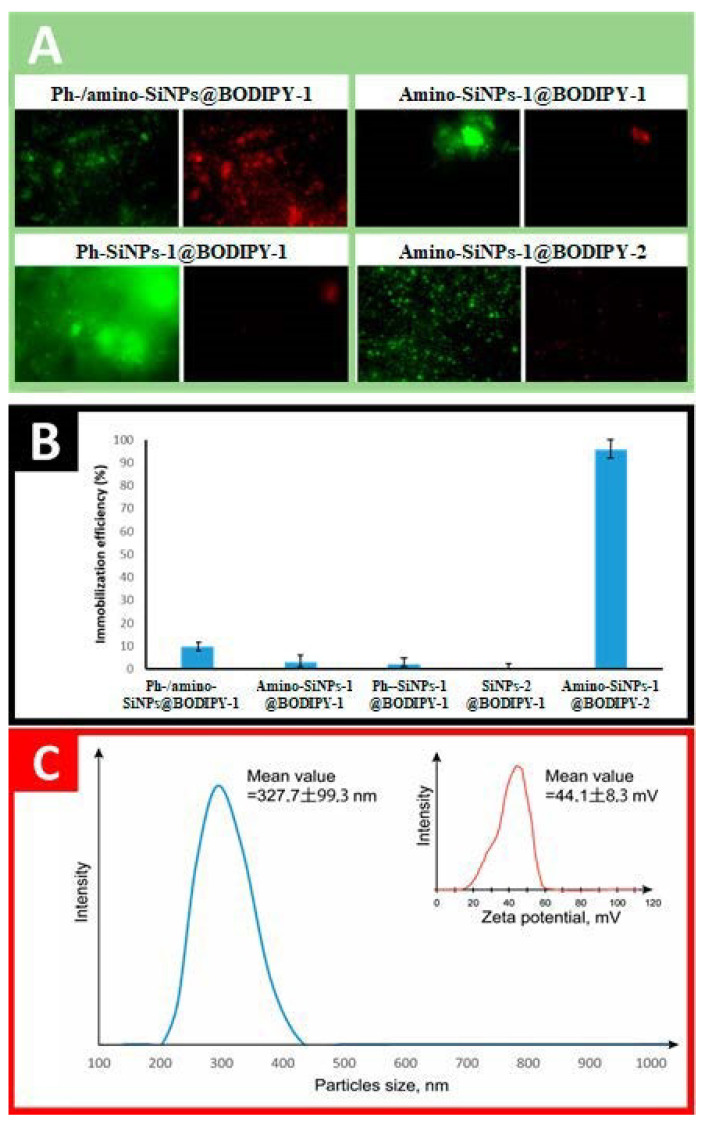
Immobilization of BODIPY-1 and BODIPY-2 onto silica-based materials. (**A**) Fluorescent images of BODIPY-conjugated silica-based materials. (**B**) The immobilization efficiency of BODIPY-1 and BODIPY-2. (**C**) Particle size distribution and zeta potential of **amino-SiNPs-1**.

Dye loading is the most important characteristic of silica-based materials and their further application as cargo carriers. The immobilization efficiency of BODIPY onto **SiNPs-1@BODIPY-1, amino-SiNPs-1@BODIPY-1** and **Ph-SiNPs-1@BODIPY-1**, **Ph-/amino-SiNPs@BODIPY-1** and **amino-SiNPs-1@BODIPY-2** were evaluated using the absorption spectra of the solution, which was obtained by dissolving silica particles containing fluorescence dye in 4% ethanolic HF solution. The immobilization efficiency of BODIPY for different tested samples is presented in Figure 7. Additionally, fluorescent microscopy at 515 (green) and 590 (red) nm was performed to observe the fluorescence of BODIPY-conjugated silica-based materials. It can be observed that the binding efficiency of BODIPY-1 depends on the type of silica-based materials. The immobilization capacity of **Ph-/amino-SiNPs@BODIPY-1** is higher than that of **SiNPs-1@BODIPY-1**, **amino-SiNPs-1@BODIPY-1** and **Ph-SiNPs-1@BODIPY-1** even though it is low (~10%). We suppose that the hydrophobic properties of phenyl groups play a vital role in the adsorption of fluorescent dye. The synergetic effect of amino- and phenyl-groups of **Ph-/amino-SiNPs@BODIPY-1** allows achieving the maximum binding efficiency for BODIPY-1 (~10%). The immobilization efficiency of BODIPY-2 in the case of **amino-SiNPs-1@BODIPY-2** was ~100%, which can be explained by covalent conjugation of BODIPY-2 with APTMS and the formation of azomethine bond (Figure 2A). To find out if BODIPY-2 was covalently incorporated into silica nanoparticles (**amino-SiNPs-1@BODIPY-2**) rather than merely physically encapsulated, we dispersed our sample in ethanol solution. Such a method for evaluation of dye leakage was previously reported in [44]. 

In parallel, we did the same procedure with other BODIPY-conjugated silica-based materials. After stirring for 24 h, dye leakage was not observed in the case of **amino-SiNPs-1@BODIPY-2**. By contrast, BODIPY release from silica particles into a dispersed medium (ethanol) was observed for other tested materials (**amino-SiNPs-1@BODIPY-1**, **Ph-SiNPs-1@BODIPY-1,** and **Ph-/amino-SiNPs@BODIPY-1**). Thus, for **amino-SiNPs-1@BODIPY-2**, the dye molecules are covalently linked to the silica matrix. We can conclude that these covalently conjugated silica nanoparticles possess advantages over physically dye-encapsulated materials due to avoiding dye leakage, which is highly important during in vitro and in vivo fluorescent imaging.

### 2.6. Cellular Uptake and Cytotoxicity Studies

**Figure 8:**
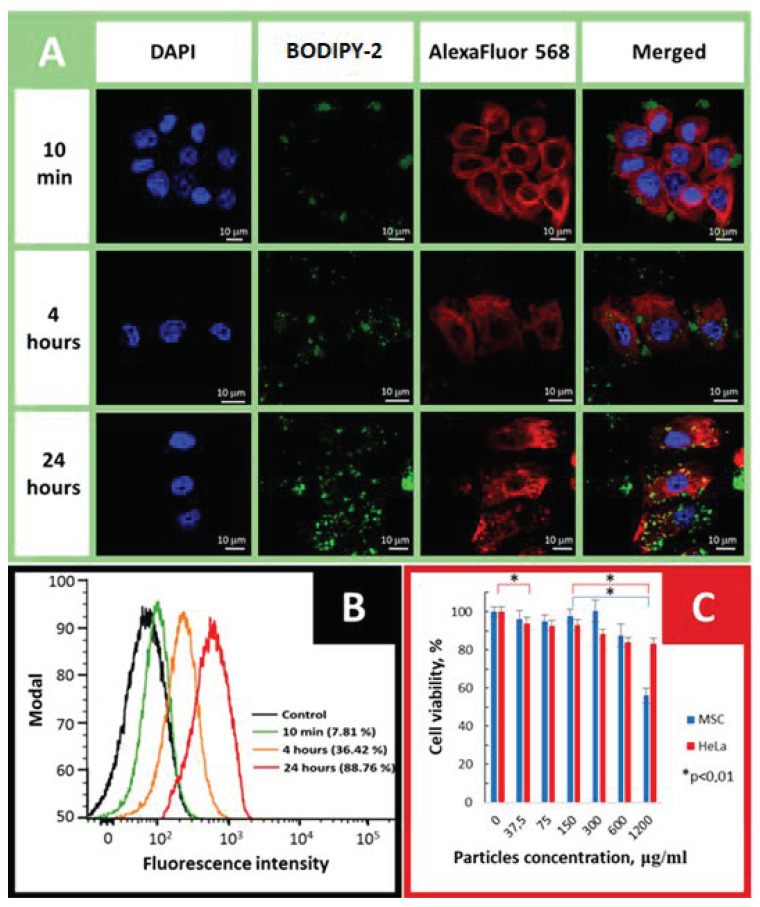
Cellular uptake efficiency and cytotoxicity studies. (**A**) CLSM images of HeLa cells treated with **amino-SiNPs-1@BODIPY-2** (concentration: 75 μg/mL) at 10 min, 4 and 24 h. (**B**) Flow cytometry analysis of HeLa cells treated with **amino-SiNPs-1@BODIPY-2** (concentration: 75 μg/mL), or medium alone (control). (**C**) In vitro cell viability of **amino-SiNPs-1@BODIPY-2** at different concentration of silica nanoparticles. The cells were treated with **amino-SiNPs-1@BODIPY-2** for 24 h. Data are presented as mean ± SD (n = 8).

Motivated by consideration of high immobilization efficiency and morphology, **amino-SiNPs-1@BODIPY-2** was selected for in vitro experiments. The human adenocarcinoma HeLa cell line was used as a cancer cell model to investigate the cellular uptake of our tested sample. To investigate the internalization of **amino-SiNPs-1@BODIPY-2,** the cultured HeLa cells were treated with 75 μg/mL of nanoparticles, followed by observation of the cells under confocal laser scanning microscopy (CLSM) at different time points (10 min, 4 h, and 24 h). As it can be seen in Figure 8A, at an initial time (t = 10 min), we did not observe cytoplasmically localized green fluorescence from BODIPY-conjugated nanoparticles. However, during the incubation period, the accumulation of **amino-SiNPs-1@BODIPY-2** nanoparticles inside cancer cells was detected and increased green fluorescence intensity originating from BODIPY-conjugated silica nanoparticles is observed (Figure 8A). The cellular uptake behavior of **amino-SiNPs-1@BODIPY-2** by HeLa cells was quantitatively studied by flow cytometry analysis. Figure 7B shows representative quantitative flow cytometry results of the cellular uptake of **amino-SiNPs-1@BODIPY-2**. As expected, the negative control showed only a low level of autofluorescence. At the same time, the uptake ratios of **amino-SiNPs-1@BODIPY-2** increased from 7.81% to 88.76% after 10 min to 24 h incubation. These results demonstrate that **amino-SiNPs-1@BODIPY-2** is readily internalized by tumorous cells. As an efficient cargo carrier, the prerequisite of **amino-SiNPs-1@BODIPY-2** should be non-cytotoxic. To evaluate the biocompatibility of our tested sample, the MTT assay presenting cell survival ratio was performed (Figure 8C). We did not observe a statistically significant reduction in the viability of the primary human mesenchymal stem cells in the particles dose range of 0–300 μg/mL as well as HeLa cells in the dose range of 37.5–150 μg/mL. For **amino-SiNPs-1@BODIPY-2,** reasonable cytotoxicity to HeLa and MSC cells was observed, and over 90% cells were showed viability even after being treated with a concentration range of 0 to 150 μg/mL of nanoparticles after 24 h incubation, indicating good biocompatibility of **amino-SiNPs-1@BODIPY-2**. It is safe to conclude that the above described high uptake efficiency of **amino-SiNPs-1@BODIPY-2** and its low cytotoxicity in this concentration is applicable for drug delivery. All these parameters indicate high potential of BODIPY-conjugated silica nanoparticles for fluorescence cell imaging.

## 3. Conclusions

In summary, we have successfully developed the new approach for obtaining BODIPY-doped silica nanoparticles via “one-pot” soft-template method using DDAO. This method allows preparing silica-based materials with definite morphology, size, structure, and functional groups. It was determined that the morphology of final silica-based materials was depended on the type of used organosilanes, the molar ratio of these organosilanes, and the presence of template (DDAO) in sol-gel synthesis. By varying the type of organosilanes, their molar ratio and using soft-template (DDAO), the most appropriate silica-based carriers for immobilization of BODIPY molecules were obtained. Different methods of BODIPY immobilization, including physical adsorption and covalent conjugation, have been applied. It was shown that the covalent conjugation of BODIPY molecules via the formation of azomethine bond provides ~100% dye immobilization without any leakage of fluorescent dye while other materials, where fluorescent dye was physically encapsulated, showed low immobilization efficiency, and the BODIPY physically encapsulated in the silica particles can easily leak out from the silica matrix. The BODIPY covalently conjugated silica nanoparticles have been chosen for in vitro experiments. These nanoparticles are efficiently uptake by tumor cells and, at the same time, they show good biocompatibility. The dye-loaded silica nanoparticles can be used for a surface modification and bioconjugation, indicating a great potential of such materials for developing the multifunctional nanoplatforms.

## 4. Materials and Methods

### 4.1. Chemicals

Tetraethoxysilane (TEOS, 99%), tetramethoxysilane (TMOS, 98%), aminopropyltrimethoxysilane (APTMS, 97%), phenyltrimethoxysilane (PhTES, 98%), dodecyl dimethylamine *N*-oxide (DDAO 30% solution in water), were purchased from Sigma-Aldrich (Germany). Cyclohexane (99%) was obtained from ECOS-1 (Russia). Absolute ethanol (C_2_H_5_OH, 98%) was used in sol-gel synthesis. Deionized (DI) water with specific resistivity higher than 18.2 MΩcm from a three-stage Milli-Q Plus 185 purification system was used. The 4,4-difluoro-8-antryl-1,3,5,7-tetramethyl-2,6-diethyl-4-boron-3a,4a-diaza-s-indacene (BODIPY-1), 4,4-difluoro-8-(3,5 dimetyl)phenyl-1,3,5,7-tetramethyl-2-formyl-4-boron-3a,4a-diaza-s-indacene (BODIPY-2) (Figure 3) were used in the experiments for their immobilization onto silica-based materials. The synthesis of these fluorescent dyes, their characterization and photophysical properties of BODIPY-1 can be found in our previously published works [55], data on BODIPY-2 are presented in [56].

### 4.2. Synthesis of Silica-Based Materials Using Sol-Gel Method 

The silica nanoparticles were prepared by the hydrolysis and polycondensation of the sol-gel precursors within the nonpolar core of oil in water emulsion. To prepare emulsion, 1.2 mL of DDAO and 0.5 mL of cyclohexane were mixed with 30 mL of water-ethanol solution (13.3% of ethanol). The resulting mixture was ultrasonicated for 5 min (ultrasonic bath 36–42 kHz, 200 W) and then was stirred (1500 rpm) during 30 min. The volume ratios of components is 6.5:1:0.125:0.3 (water:ethanol:cyclohexane:DDAO 30% solution)

Different types of organotrialkoxysilane precursors were added at various molar ratios to the formed emulsion. Sol-gel precursors: TEOS, TMOS, APTMS, and PhTES were used in the synthesis. The details for synthetic procedures of silica-based materials are presented in Supplementary Materials. At the first stage two sol-gel precursors were added to the reaction mixture and the resulting solution was stirred for 10 min at 60 °C. After that, the next precursor was added and the resulting mixture was stirred for 24 h at room temperature (20 °C). Then a viscous stable colloid of white color was formed, a vacuum filtration method was used (a Bunsen flask with a Schott filter) to separate the dispersed phase from the dispersion medium. The solid phase was washed with a water-ethanol solution twice and dried under vacuum at 95 °C for 2 days. The synthesis of silica-based materials was performed using different sol-gel precursors, varying their molar ratio, with and without co-template (cyclohexane/DDAO) (see Supplementary Materials) in order to detect the influence of these factors on the morphological structure of final materials scanning electron microscopy was used. Then the most appropriate samples have been chosen for immobilization of BODIPY.

### 4.3. Immobilization of BODIPY-1 and BODIPY-2 Into Silica-Based Materials 

BODIPY-1 and BODIPY-2 (Figure 8) were used to investigate the possibility of immobilization by noncovalent and covalent ways. The BODIPY-1 was injected in reaction mixture by dissolving in organosilane precursors as TMOS, APTMS, PhTES (see Section 2.2. **Synthesis of silica-based materials using sol-gel method**), quantity of dye was 4.5·10^−8^ mol (determined by absorption spectra).

For immobilization of BODIPY-2, the solution contained 4.5·10^−8^ (determined by absorption spectra) mol of dye and APTMS (220 μL) was prepared. Moreover, 20 μL of the resulting solution was dissolved in 980 μL of ethanol for evaluation of conjugation process. The remaining 200 μL of solution were stirred for 24 h at room temperature in order to obtain a sol-gel precursor conjugated with BODIPY-2 (APTMS@BODIPY-2). The concentration and reaction ending were additionally approved with absorption spectra. Subsequently, this modified precursor was used in the similar synthesis instead of native APTMS for synthesis of **amino-SiNPs-1@BODIPY-2**.

The amount of immobilized BODIPY molecules onto the silica matrix was evaluated by dissolving silica particles containing fluorescence dye in a 4% (*v*/*v*) HF in ethanol. After that, the obtained solutions were collected and amounts of BODIPY were quantified from UV-Vis absorption spectra. The immobilization efficiency was calculated as ratio of final concentration of BODIPY after dissolution of silica in 4% ethanolic HF and initial concentration of BODIPY used for immobilization.

### 4.4. Samples Characterization

#### 4.4.1. Scanning Electron Microscopy (SEM) 

The morphology of obtained silica-based materials was observed on VEGA 3 Scanning Electron Microscope (SEM “TESKAN”, Czech Republic) at an accelerating voltage of 5 kV. The samples were sprayed onto the carbon scotch.

#### 4.4.2. Fourier Transformed Infrared Spectroscopy (FTIR) 

FTIR spectra of obtained samples were recorded on a spectrometer Avatar 360 (“Thermo Nicolet”, USA). The spectra were performed in the range of 400–4000 cm^−1^ at room temperature. The transmittance measurements were carried out in a KBr pellet.

#### 4.4.3. Fluorescence Microscopy

Fluorescence microscopy was performed using Lyum 3 microscope (“Micromed”, Russia) under the ×60 magnification, in two modes: (a) B filter—the excitation wavelength *λ*_ex_ = 410–490 nm and 515 nm barrier filter (b) G filter—*λ*_ex_ = 500–550 nm and 590 nm barrier filter. Pictures were registered using TopupCam UCMOS 3 mp (camera) controlled from PC via ToupView software. The interaction of cells with nanoparticles was observed using Confocal Laser Scanning Microscopy (CLSM, Leica TCS SP8 confocal microscope, Germany) with 40× high numerical-aperture oil immersion objectives.

#### 4.4.4. UV–Vis and Fluorescence Spectra

UV–Vis electronic absorption spectra (EAS) of solutions were recorded in the range 420–600 nm using an SF-104 spectrophotometer (“Aquilon”, Russia) controlled with a PC through the UVWin 5.1.0 software package. The wavelength accuracy was ±0.05 nm. Investigations of solutions were carried out in quartz cuvettes with a thickness of the absorbing layer of 10 mm. 

Fluorescent spectra of solutions and films were obtained with a Cary Eclipse fluorescence spectrometer (“Varian-Agilent”, US-Australia) controlled with a PC using the Cary Eclipse Scan Application 1.1 software package in the wavelength range 490–600 nm with 2.5 nm excitation and emission slit widths. The excitation wavelength was 480 nm. 

### 4.5. Cell Culture

Human cervical cancer cell line (HeLa) was provided by Institute of Cytology of Russian Academy of Sciences. Cells were cultured in Dulbecco’s Modified Eagle Medium (DMEM) (Lonza, Switzerland); supplemented with 100 IU/mL penicillin, 0.1 mg/mL streptomycin (Biolot, Russia), and 10% Fetal Bovine Serum (FBS) (HyClone, USA). After achievement of confluence (>80%), cells were detached with trypsin/EDTA solution (Invitrogen, USA) for 2 min and seeded to attach at a density of 5.0 × 10^3^ cells/cm^2^. Human mesenchymal stem cells (MSCs) were derived from the bone marrow sample of healthy donors who had signed informed consent. The experiments involving donor material were approved by Pavlov University institutional review board. Cells were isolated using direct plating procedure as follows: 1 mL of heparinized whole bone marrow was resuspended in Minimum Essential Medium (alpha-MEM) (Lonza, Switzerland); supplemented with 100 IU mL^−1^ penicillin, 0.1 mg mL^−1^ streptomycin (Biolot, Russia), and 10% of Fetal Bovine Serum (FBS) (Hyclone, USA). After achievement of 80% confluence, cells were detached by trypsin/EDTA solution (Invitrogen, USA) for 5 min and re-plated/passaged at a density of 5.0 × 10^3^ cells cm^−2^. Cultures of 1 to 2 d passages were used for experiments.

### 4.6. Incubation of HeLa Cells with BODIPY-Conjugated Silica Particles

HeLa cells were seeded on glass coverslips in a 12-well culture plate and kept under standard cell culture condition. After 24 h, the solution of BODIPY-conjugated silica particles (**amino-SiNPs-1@BODIPY-2**) at concentration of 100 µg/mL. At definite time points, the cells were fixed at 4% of paraformaldehyde solution, permeabilized (0.1% Triton), and viewed under CLSM. The cells were fixed in 4% solution of paraformaldehyde in PBS. Then they were permeabilized by 0.1% PBS-buffered Triton X100 for 5 min, and stained with 4’,6-diamidino-2-phenylindole (DAPI) and Alexa Flour 568 Phalloidin (AF 568 PL). The uptake behavior of BODIPY-conjugated silica particles (**amino-SiNPs-1@BODIPY-2**) by HeLa was evaluated using flow cytometry (Accuri C6 Cytometer). For this reason, the silica particles (100 µg/mL) were incubated with HeLa cells and MSCs at 37 ^o^C under 5% CO_2_ atmosphere for 3 and 6 h. After incubation, the cells were washed with PBS, detached by trypsin, and centrifuged at 1400 rpm for 5 min. Then, cells were resuspended in 0.5 mL of PBS and analyzed by Accuri C6 Cytometer. 

### 4.7. Cell Viability Assay

BODIPY-conjugated silica particles (**amino-SiNPs-1@BODIPY-2**) at different concentrations (1200 µg/mL, 600 µg/mL, 300 µg/mL, 150 µg/mL, 75 µg/mL, 32.5 µg/mL) were added into the wells of 96-well plates with confluent monolayer of HeLa/MSC’s (104 cells/mL) in 8 repeats. Cells were incubated for 24 h at 37 °C in a 5% CO_2_ atmosphere. For evaluation of cytotoxicity of particles MTT (3-(4,5-Dimethylthiazol-2-yl)-2,5-Diphenyltetrazolium Bromide (5 mg/mL, 100 µL on well) was performed. After that the purple formazan crystals were dissolved by DMSO, the absorbance of the wells at 490 nm were measured with a microplate reader with base line by non-treated wells.

### 4.8. Statistics

Statistical analyses were performed using Prism5 (Graph Pad, La Jolla, CA, USA) and SPSS (IBM, USA) software. The descriptive statistics methods were applied when appropriate. All values were plotted as averages with standard deviations of the means. The statistical significance of the difference in the viability according to the silica particles dose were analyzed using analysis of variance (ANOVA). The significance level was defined as *p* < 0.05.

## Supplementary Materials

The following are available online: Table S1.
