# Supplementary material for: DDAO Controlled Synthesis of Organo-Modified Silica Nanoparticles with Encapsulated Fluorescent Boron Dipyrrins and Study of Their Uptake by Cancerous Cells
Source: Molecules. 2020 Aug 21;25(17):3802. doi: 10.3390/molecules25173802 (PMC7504138; doi:10.3390/molecules25173802)
Supplement: Supplementary file 1 [file molecules-25-03802-s001.pdf]

Table S1. Synthetic systems numeration and the amounts of used chemicals.

№	Material name	Compounds of reaction environment	Sol-Gel precursor
1	**SiNPs-1**	*Solv + **Template	TEOS 0.7 ml
2	**SiNPs-2**	Solv + Template	TEOS 0.6 ml + TMOS 0.1 ml
3	**amino-SiNPs-1**	Solv + Template	TEOS 0.5 ml + APTMS 0.2 ml
4	**Ph-SiNPs-1**	Solv + Template	TEOS 0.5 ml + PhTEOS 0.2 ml
5	**SiNPs-3**	Solv	TEOS 0.7 ml
6	**SiNPs-4**	Solv	TEOS 0.6 ml + TMOS 0.1 ml
7	**amino-SiNPs-2**	Solv	TEOS 0.5 ml + APTMS 0.2 ml
8	**Ph-SiNPs-2**	Solv	TEOS 0.5 ml + PhTEOS 0.2 ml
9	**Ph-/amino-SiNPs@Bodipy-1**	Solv + Template + BODIPY1	TEOS 0.4 ml + PhTEOS 0.1 ml + APTMOS 0.2 ml
10	**amino-SiNPs-1@Bodipy-1**	Solv + Template + BODIPY1	TEOS 0.5 ml + APTMS 0.2 ml
11	**Ph-SiNPs-1@Bodipy-1**	Solv + Template + BODIPY1	TEOS 0.5 ml + PhTEOS 0.1 ml + TMOS 0.1 ml
12	**SiNPs-1@Bodipy-1**	Solv + Template + BODIPY1	TEOS 0.6 ml + TMOS 0.1 ml
13	**amino-SiNPs-1@Bodipy-2**	Solv + Template	TEOS 0.5 ml + APTMS-BODIPY2 conjugate 0.2 ml

*Solv = H_2O (26 ml) + C_2H_5OH (4 ml)

**Template = DDAO (1.3 ml) + cyclohexane (0.5 ml)
